# Effect of Motor versus Sensory Nerve Autografts on Regeneration and Functional Outcomes of Rat Facial Nerve Reconstruction

**DOI:** 10.1038/s41598-019-44342-9

**Published:** 2019-06-07

**Authors:** S. Ahmed Ali, Andrew J. Rosko, John E. Hanks, Aaron W. Stebbins, Osama Alkhalili, Norman D. Hogikyan, Eva L. Feldman, Michael J. Brenner

**Affiliations:** 1 Department of Otolaryngology – Head and Neck Surgery, Michigan Medicine, Ann Arbor, MI USA; 2 Department of Neurology, Michigan Medicine, Ann Arbor, MI USA

**Keywords:** Regeneration and repair in the nervous system, Somatic system

## Abstract

Cranial nerve injury is disabling for patients, and facial nerve injury is particularly debilitating due to combined functional impairment and disfigurement. The most widely accepted approaches for reconstructing nerve gap injuries involve using sensory nerve grafts to bridge the nerve defect. Prior work on preferential motor reinnervation suggests, however, that motor pathways may preferentially support motoneuron regeneration after nerve injury. The effect of motor versus sensory nerve grafting after facial nerve injury has not been previously investigated. Insights into outcomes of motor versus sensory grafting may improve understanding and clinical treatment of facial nerve paralysis, mitigating facial asymmetry, aberrant reinnervation, and synkinesis. This study examined motor versus sensory grafting of the facial nerve to investigate effect of pathway on regeneration across a 5-mm rodent facial nerve defect. We enrolled 18 rats in 3 cohorts (motor, sensory, and defect) and recorded outcome measures including fiber count/nerve density, muscle endplate reinnervation, compound muscle action potential, and functional whisker twitch analysis. Outcomes were similar for motor versus sensory groups, suggesting similar ability of sensory and motor grafts to support regeneration in a clinically relevant model of facial nerve injury.

## Introduction

Cranial nerve injury after head and neck surgery can have disabling functional consequences. Two of the most commonly injured nerves are the facial nerve (CN VII) and the recurrent laryngeal nerve (RLN, CN X). These injuries may arise from congenital, infectious, idiopathic, traumatic, neoplastic, endocrine, neurologic, and systemic causes. It is also not uncommon for nerve to be deliberately sacrificed at time of surgery in order to completely remove malignancy. Facial nerve injury results in oral incompetence, dysarthria, visual impairment, facial asymmetry, and synkinesis, in which miswiring of nerves results in synchronous involuntary muscle movement with expression^1,2^. Recurrent laryngeal nerve injury may result in altered voice, dysphagia, and pneumonia secondary to aspiration; these complications may necessitate invasive procedural support, feeding tube usage, and surgical airway (tracheostomy) placement^3^.

In the case of simple transection of a peripheral nerve, the gold standard remains end-to-end re-approximation via microsurgical neurorrhaphy^4^. Ablative surgery for cancer may necessitate resection of the entire nerve or peripheral nerve branches^5^. If the nerve gap is larger than 5-mm, then primary repair may be suboptimal, as excess tension reduces axonal diameter, places ischemic stress on the nerve, and is associated with worse functional outcomes^6–8^. Thus, nerve interposition or “cable graft” procedures are commonly performed clinically to bridge larger nerve gaps. While the graft should have similar cross-sectional area to the recipient nerve, additional length or altered geometry of the autograft does not appear to have an influence^9^. Reversing polarity of the cable graft has been theorized to influence nerve regeneration, however, this has not borne out in animal models through functional, histologic, or electrophysiologic parameters^10–13^.

For head and neck procedures, clinicians have historically utilized a variety of donor sites to obtain donor nerves for grafting. Common options include the ansa cervicalis nerve, greater auricular nerve, sural nerve, medial antebrachial cutaneous nerve, and nerve to the vastus lateralis. Given lack of full recovery of the transected or resected nerve after reconstruction of the nerve defect, there remains significant room for improvement through scientific experimentation^14^. Current considerations for selecting an autograft include donor site morbidity (loss of sensation, pain, paresthesia, or loss of motor function arising from removal of a nerve for use in grafting), branching pattern, length of nerve required to reconstruct the defect, caliber-match of the nerve, ease of harvest, and need for incisions^15^.

An additional consideration is whether the donor nerve is of predominantly a motor versus sensory origin. Clinically, the nerves utilized are almost exclusively sensory nerves. This practice is at odds with several animal studies finding that after injury motor axons will preferentially reinnervate motor versus sensory pathways^16,17^. Studies have demonstrated improved nerve regeneration seen with motor autografts in comparison to sensory grafts in terms of nerve fiber count, percent nerve, and nerve density^18,19^. These prior studies were conducted in a femoral or tibial nerve model and may not be applicable to the facial nerve model, which is a cranial nerve with distinct functional characteristics. There has been growing interest in identifying the optimal type of graft. The present study investigated whether use of motor nerve autografts improved neuroregeneration relative to sensory autografts in the rat facial nerve, based on electrophysiologic, histomorphometric, and functional parameters.

## Results

All animals enrolled in the study survived to the 15-week end-point. The rats gained weight appropriately throughout the study period; there were no untoward effects from surgery or from repeat electrophysiologic data collection. There was appropriate wound healing over the surgical site following initial microsurgery.

### Histology

**Figure 1:**
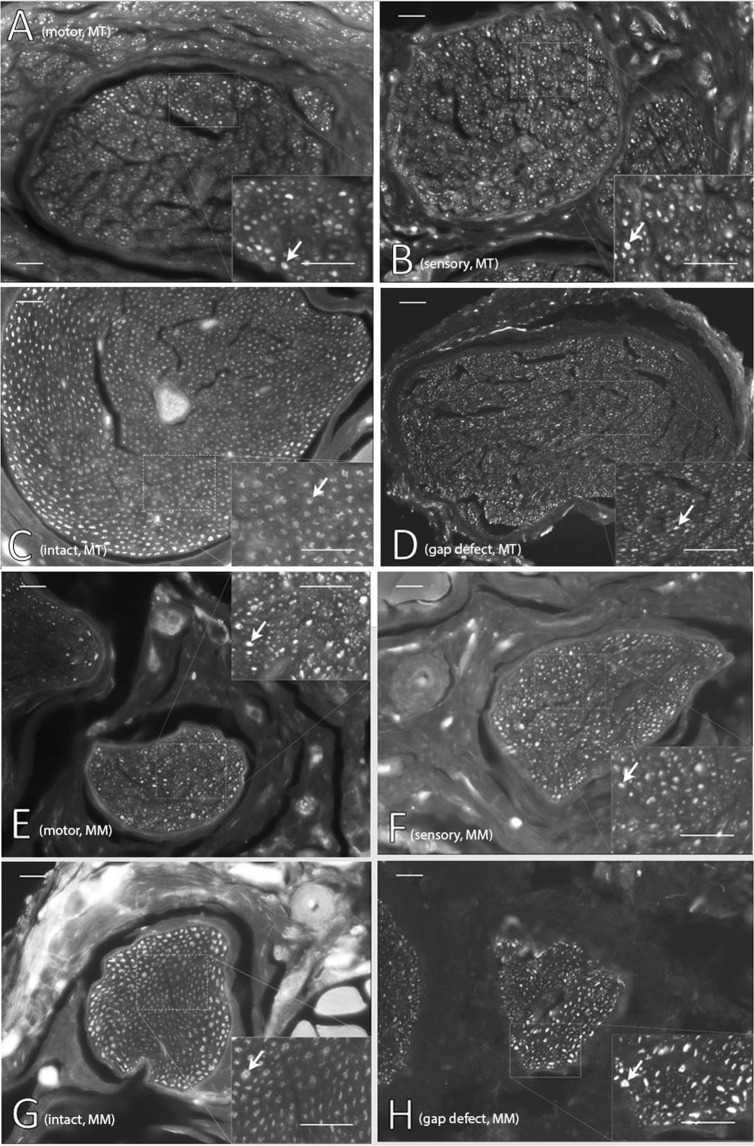
Representative cross-sectional histology of nerve specimen stained with neurofilament from final endpoint of 15 weeks. White arrows denote evidence of regenerating nerve fiber. Facial nerve main trunk (MT) is demonstrated for **(A)** motor isograft group, **(B)** sensory isograft group, **(C)** intact group, and **(D)** gap defect group. Marginal mandibular (MM) nerve histology is demonstrated for **(E)** motor isograft group, **(F)** sensory isograft group, **(G)** intact group, and **(H)** gap defect group. Main image is imaged at 20X magnification and representative inset images are imaged at 40X magnification. Scale bars are 200 µm uniformly.

Qualitative analysis of cross-sections of the peripheral marginal mandibular branch and main facial trunk demonstrated robust regeneration for the motor and sensory isograft groups at the conclusion of the study. Representative histology for the motor, sensory, uninjured, and gap defect groups are demonstrated in Fig. 1.

**Figure 2:**
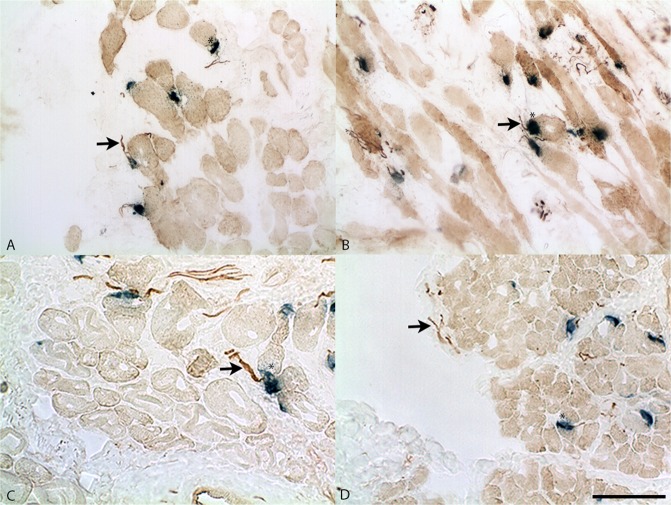
Representative histology of muscle specimen imaged via light microscopy at 20X magnification. Vibrissal pad muscle is demonstrated for **(A)** motor isograft group, **(B)** sensory isograft group, **(C)** intact group, and **(D)** gap defect group. The background is light brown, nerve fibers are dark brown (neurofilament stain), and motor endplates (acetylcholinesterase stain) are stained blue. Asterisk represents motor end-plates and black arrow points to nerve fibers. Scale bar represents 100 µm.

Qualitative analysis of cross-sections of vibrissal motor end plates demonstrated similar extent of innervated end plates between the motor and sensory groups. Representative images used for analysis are demonstrated in Fig. 2.

### Histomorphometry

**Figure 3:**
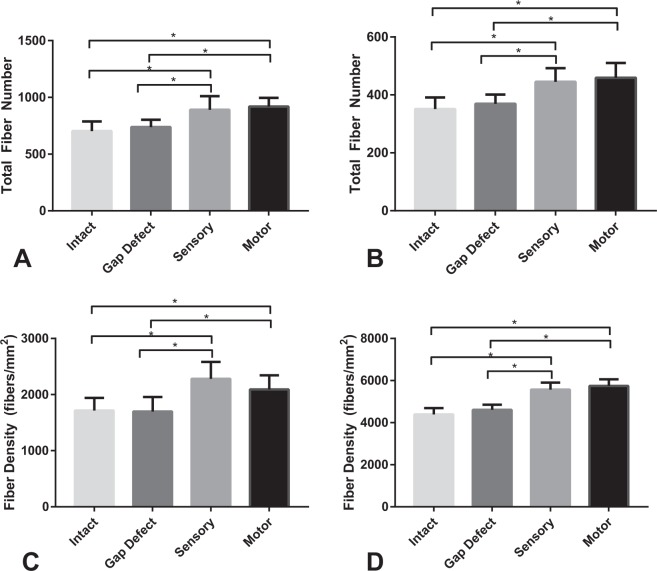
Histomorphometric data representing **(A)** main trunk total fiber count, **(B)** marginal mandibular total fiber count, **(C)** main trunk never density and **(D)** marginal mandibular nerve density. The motor isograft and sensory isograft groups demonstrated consistently greater total fiber count and nerve density measures in comparison to the gap defect and intact groups for both the main trunk and marginal mandibular nerve measures (*p < 0.05, student’s t-test).

Our group performed quantitative histomorphometric analysis of the facial nerve trunk, including nerve fiber counts and fiber density (measured in fibers/mm^2^). These data are modeled for the main trunk and marginal mandibular nerve cohorts in Fig. 3, Tables 1 and 2. At the 15-week end-point, robust nerve regeneration was substantial for all 3 intervention groups (motor autograft, sensory autograft, and gap defect group). There was similarity in nerve density measurements as well as nerve fiber counts between the motor and sensory groups; total fiber counts of 919 (SD 76) versus 891 (SD 120) respectively and fiber density 2,090 fibers/mm^2^ (SD 254) versus 2,280 (SD 300) for the main trunk measurement (*P* = 0.64 for total fiber count and *P* = 0.26 for fiber density). Nerve density and total fibers counts for the experimental groups were consistently greater compared to the uninjured group and the gap defect group (all *P* < 0.05). Total fiber counts and nerve density measurements were not statistically significant between any of the groups for the marginal branch (all *P* > 0.05).

**Table 1:** Total fiber numbers and fiber density for the main trunk.

Group	Total Number Fibers (mean count, SD)	Fiber Density (mean fibers/mm^2^, SD)
Motor	919 (76)	2090 (254)
Sensory	891 (120)	2280 (300)
Uninjured	703 (85)	1715 (226)
Gap defect	738 (65)	1699 (256)

**Table 2:** Total fiber numbers and fiber density for the marginal mandibular branch.

Group	Total Number Fibers (mean count, SD)	Fiber Density (mean fibers/mm^2^, SD)
Motor	459 (51)	5743 (316)
Sensory	445 (47)	5568 (335)
Uninjured	351 (40)	4393 (294)
Gap defect	369 (32)	4612 (237)

### Electrophysiological analysis

**Figure 4:**
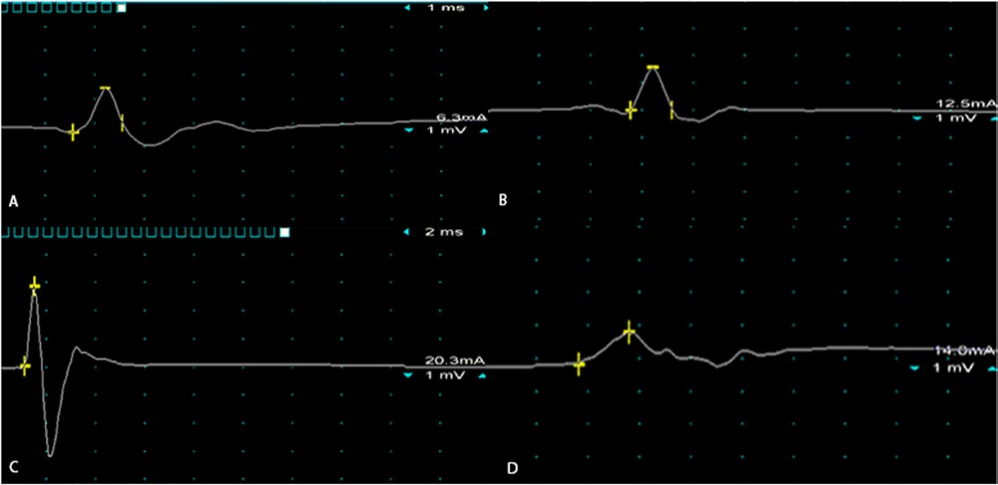
Representative electrophysiological tracings from **(A)** motor isograft, **(B)** sensory isograft, **(C)** intact, and **(D)** gap defect groups at week 15 of the experiment. The yellow bars in the tracings represent marks for measuring amplitude (in millivolts) from baseline.

**Figure 5:**
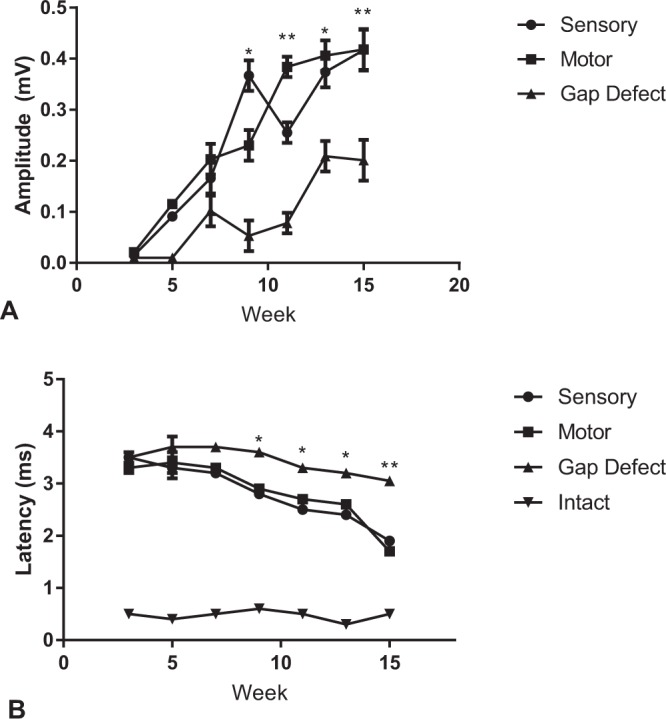
Electrophysiological compound muscle action potential data (**A**) and latency data (**B**) for the experimental groups. There was no significant difference in motor versus sensory autograft trends throughout the experimental timeline. From the week 9 mark and forward, mean amplitude in millivolts (mV) and mean latency in milliseconds (ms) were greater for both the motor and sensory groups in comparison to the control group (*p < 0.05, **p < 0.005, student’s t-test).

Representative tracings from the groups are displayed in Fig. 4. Compound muscle action potentials measured at 3, 5, 7, 9, 11, and 13 weeks are shown in Fig. 5. There were similar trends for the motor and sensory autograft groups; aside from discrepancy of the mean amplitude ratio at the 9-week mark (all *P* > 0.05). In comparison to the gap group, at the final endpoint the mean amplitude ratio was greater for the motor group (0.417 vs 0.201, *P* = 0.003) and the sensory group (0.418 vs 0.201, *P* = 0.003).

### Functional whisker pad analysis

**Figure 6:**
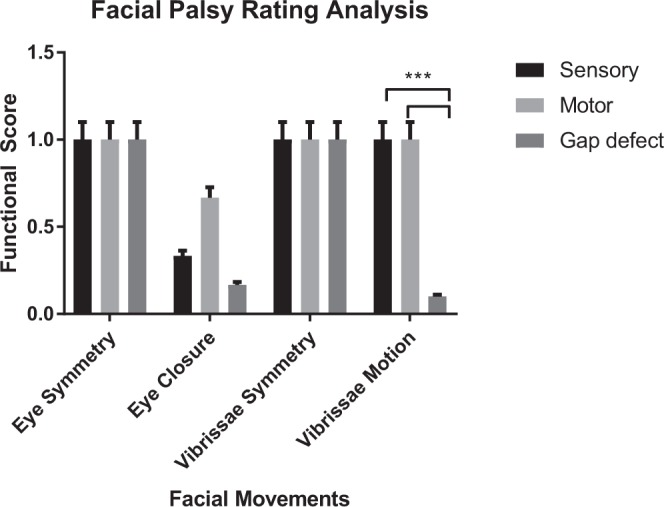
Rat facial palsy scores for week 13 endpoint across experimental groups. There was no statistically significant difference in mean scores when comparing motor versus sensory groups. Vibrissal motion scores were significantly greater for the motor and sensory groups in comparison to the control group (***p < 0.0001, student’s t-test).

Rat facial palsy scores for the experimental groups are modeled in Fig. 6. At the final endpoint, all animals demonstrated return of muscle tone of their whisker pad, as they scored a 1 on the facial palsy scale. The motor and sensory groups demonstrated similar scores for the eye symmetry and vibrissal motion. The motor group demonstrated a numerically higher mean score for eye closure than the sensory group, although this difference was not statistically significant (0.66 versus 0.33, *P* = 0.37). Overall, there were no statistically significant differences between mean scores for the groups. Both the motor and sensory groups demonstrated significantly greater scores for vibrissal motion than the control (1.0 versus 0.1, *P* < 0.0001).

## Discussion

This study found no significant differences in outcomes arising from motor versus sensory grafting for reconstruction of a rodent main trunk facial nerve gap injury. The outcomes of motor versus sensory grafting were comparable across all 4 validated outcome measures, including electrophysiological compound muscle action potential testing, functional whisker twitch analysis, qualitative and quantitative nerve analysis. These findings support the current surgical reconstructive practice of utilizing sensory nerves to bridge peripheral motor nerve defects.

The question of whether motor isografts could confer a better outcome is an important one given the morbidity associated with facial nerve injury and inherent limitations in functional outcome after grafting. In general, the optimal result patients can hope to achieve after reconstruction of a transection or nerve gap injury is a House-Brackmann grade III score after microsurgical repair^20^. Previous *in vitro* work has demonstrated potential preferential reinnervation of motor pathways versus sensory pathways after peripheral nerve injury^16,17,21,22^. From an architectural perspective, motor nerves contain a greater number of large axons, greater fiber width, heavier myelination, and a lower fiber density than sensory nerves^23,24^. The notion that motor autografts may be more conducive to regeneration than sensory autografts is further bolstered by neurotropic and neurotrophic considerations. There is a process of preferential motor reinnervation (PMR) secondary to motor axons receiving positive trophic support as they regrow, versus sensory nerves that are essentially “starved” as they grow in a mismatched pathway^18^. Phenotypic studies evaluating axonal regeneration have demonstrated that Schwann cells express distinct sensory and motor phenotypes that support regenerative capacity in a phenotype-specific manner^25^. These data would lead one to expect differential regenerative outcomes as a function of graft type.

There are conflicting findings on the application of PMR to nerve grafting studies. In separate studies in the rat tibial nerve model, histomorphometric analysis at the 3-week mark demonstrated poorer nerve regeneration in the sensory group in comparison to the motor group^18,19^. Nichols and colleagues demonstrated that improved nerve regeneration was seen with motor autografts in comparison to sensory grafts in terms of nerve fiber count, percent nerve, and nerve density in the tibial nerve model^18^. In a follow-up study, also in the tibial nerve model, we demonstrated significantly improved regeneration independent of graft cross-sectional area or cable number across similar parameters^19^. A subsequent study demonstrated no differences at the 3-week time-point in analyzing myelinated axon counts between sensory, motor, and mixed (both motor and sensory components) autografts in a mixed nerve defect model^26^. Similarly, a large-scale study in 100 rats demonstrated equivocal results in motor, sensory, mixed nerve grafts via histomorphometric analysis at 5, 6, and 7 weeks in the tibial nerve model^27^. A potential shortcoming to the studies that demonstrated motor isograft superiority is the early endpoint, with histomorphometric analyses at three weeks after reconstruction^18,19^. Studies evaluating nerve regeneration following various mechanisms of injury have demonstrated continued evidence of evolution of regeneration for 9–12 weeks after initial insult^28,29^.

One potential contributor to the discrepant findings in the literature is the exceptional regenerative potential observed in the rat. In this animal model, some studies report that the advancing nerve front will bypass the distal injury site even in negative controls, rendering treatment effects across treatment groups histologically indistinguishable^30^. The treatment effect appeared to plateau at the 70-day mark in the aforementioned study^30^. Despite this apparent limitation, the study by Kawamura *et al*. did not demonstrate histomorphometric difference at 5-weeks, leading us to believe that the lack of treatment effect cannot be solely explained by this phenomenon^27^. The vibrissal pad and electrophysiological analyses conducted over the experimental timeline demonstrated similar outcomes for motor and sensory groups.

When assessing the results of neurofilament staining of nerve specimens (Fig. 1), qualitatively our group noted scattered and unorganized regenerating nerve fibers in the gap defect in comparison to the motor and sensory isograft groups. As expected, the intact nerve group demonstrated uniform staining and organization of nerve fibers. In analyzing the histomorphometric data, our analyses consistently demonstrated greater motor and sensory isograft fiber counts in comparison to the gap defect group. Interestingly, these two groups also demonstrated significantly greater fiber counts in comparison to the intact nerve group. As injured nerves regenerate, especially in rodents that demonstrate excellent regenerative potential, axonal sprouting occurs, and there exists the potential for the total number of nerve fibers of previously injured nerves to bypass even healthy controls, a phenomenon that may be related to the structure of the peripheral nerve graft^31,32^. A similar finding may be occurring in rats with short gap defect injuries^32^. Whereas studies have demonstrated significantly different axonal counts in longer gap defects (>5 mm), they have found similar nerve counts between gap defect and normal nerves in short defects^3,33^. Previous literature also demonstrates that axonal sprouts distal to injury may outnumber those in the proximal stump at a later timepoint^34^. This phenomenon of potential axonal sprouting after injury must be viewed in conjunction with measures of functional recovery, which is the ultimate test of nerve regeneration^35^. Herein lies the importance of the electrophysiological data. These data demonstrate superior results for the motor and sensory isograft groups compared to the gap defect group from the week 9 time point till the conclusion of the experiment^36^. This finding correlates with the superior whisking movement of the motor and sensory isograft groups in comparison to the gap defect group.

We are unaware of studies comparing regeneration in peripheral versus cranial nerve injury models, and the role of grafting has been primarily emphasized in extremity nerve injury models. The benefits of a facial nerve model are two-fold: (1) it allows comparison of previous histomorphometric data to the facial nerve model with additional information from CMAP and whisker analysis, and (2) it may afford an opportunity for assessing synkinetic or aberrant regeneration of spinal nerves via the unique facial nerve model. There are specific factors unique to the facial nerve model to consider in comparison to other clinical nerve impairments. When assessing response of treatment or procedural intervention, the overall regenerative, reinnervation, and movement outcomes are the most important parameters to consider as has been emphasized by numerous landmark studies in the literature over time^37–39^. The movement-related outcomes are represented in the grading schemes for characterizing clinical facial nerve injury, and ultimately are a direct reflection of extratemporal facial nerve motor regeneration^30,40^. From a patient-centered perspective, facial expression, tone, and movement are foremost concerns^41,42^. From a reconstructive standpoint, current and emerging approaches to facial nerve rehabilitation emphasize recovery of facial expression, tone, and movement^43–45^.

The facial nerve rodent model is limited to an approximately 5-mm nerve gap, due to the short distance from the skull base to the initial *pes anserinus*. This is an important consideration when considering extrapolating such findings to larger defects in human patients. An additional challenge in translating such findings to clinical practice is the heterogeneity in nerve injury (location, length, involvement of branching) and host characteristics (history of prior radiation, diabetes, other wound healing factors). Given the findings in this study – that motor and sensory isografts demonstrate equivalent results across several parameters – and the additional morbidity associated with harvest of motor grafts, it would be prudent for clinicians to continue current clinical practice of harvesting sensory autografts for nerve defect repair.

### Limitations

Quantification with confocal microscopy and Z stack series analyses is required to assess exact numbers of motor endplates^46,47^. In the current study, histomorphometric analysis of the vibrissal pad muscle via light microscopy produced qualitative images confirming the presence of motor endplates however did not allow for exact quantification^48^. There were additional limitations with implementation of the facial palsy rating analysis to our cohort. The videographic analysis was performed at week 15, a time point at which even unrepaired gap defect injuries have clear evidence of regeneration^28^. The measures of eye and vibrissal symmetry had normalized between groups by this point. Eye closure was difficult to assess across groups without presence of a consistent stimulus to test this function. In our experience, the degree of vibrissal motion was consistently different between the intervention groups and negative control. A more refined assessment of function should be considered for future experiments, including utilization of olfactory and whisking stimuli^40^. Lastly, interpretation of histomorphometry of the gap defect group is limited by lack of supporting contextual data. While previous studies have demonstrated evidence of axonal sprouting in short gap defect models, there exists little supporting evidence of sprouting in main trunk gap defect facial nerve models^33^. Although it is not possible to introduce a critical defect (1 cm) in the rat main trunk facial nerve model, future studies could better elucidate sprouting in gap defects in other animal models or in more peripheral branches of the rat facial nerve.

## Conclusion

Autologous nerve grafting is the gold standard for clinical repair of large-gap defects of the facial nerve. Numerous *in vitro* studies demonstrate preferential motor reinnervation, raising the possibility of improved clinical outcomes with motor nerve cable grafting. While the experimental data on motor nerve grafts in the rat sciatic nerve model has have been suggestive of a benefit to motor grafting, no difference between motor and sensory graft cohorts was observed in this model of facial nerve gap reconstruction, based on histological, electrophysiological, and functional outcome parameters. Further studies with larger cohorts in a large animal model with serial outcome measures are needed. Given early exploration into motor grafting of nerve defects in patients, there is also a potential role for clinical studies to investigate whether findings of animal studies are borne out by clinical experience^49^.

## Materials and Methods

### Animal housing

All interventions were performed in strict accordance with the National Institutes of Health guidelines. The experimental protocol was approved by the University of Michigan Institutional Animal Care & Use Committee (IACUC) prior to implementation of the protocol (PRO00008431). Adult Sprague-Dawley rats weighing 250 to 350 g were quarantined and housed in a central animal care facility prior to initiation of the experimental protocol. All animals were provided a 12-hour light/dark cycle. Food and water were available *ad libitum*. After procedures, the animals were recovered in a warm environment underneath a heat lamp and closely monitored for 3 hours. Animals were then returned to the central animal care facility. The animals were monitored twice daily for the first three post-operative days for signs of weight loss, infection, wound breakdown, excessive pain, or other morbidity. They were thereafter monitored once daily for 1 week by members of the surgical team and the veterinary care team.

### Animal treatment and experimental design

A total of 18 rats were randomized into 3 groups of 6 rats each. In all of the animals, a 5-mm nerve gap was created in the animal’s left facial nerve main trunk. For the experimental groups, Group I consisted of rats who had their nerve gap defect repaired with a motor autograft harvested from the femoral branch to the quadriceps. Group II consisted of rats who had their nerve gap defect repaired with a sensory autograft harvested from the femoral saphenous branch. Group III consisted of our positive control group, and no repair was performed for their nerve gap defect. The non-operated right face of the rat served as the negative controls.

### Surgical techniques

A dedicated procedure room was utilized for surgical procedures. General anesthesia in the animal was induced via 1.8% isoflurane. Following induction of general anesthesia, maintenance was continued via nose-cone; flow of the inhaled anesthetic and oxygen was monitored and adjusted as necessary by an assistant. A heated water conductor pad (Aqua Relief System, PMT, Akron, OH) was fixed underneath the sterile operating table drape to regulate body temperature. Petrolatum eye gel was applied bilaterally to guard against corneal irritation or dryness. An ear tag was applied to the animal and the operative sites were shaved, prepped with chlorhexidine and sterile water and draped in accordance with proper aseptic technique. The animal’s wakefulness was tested via hind paw toe-pinch. A subcutaneous injection of 0.05 mg/kg of buprenorphine was administered. Sterilized instruments were used for the dissection. An operating microscope was utilized for the entirety of the surgical procedure (Wilde M690, Leica Microsystems, Switzerland).

For Group 1 and Group 2 animals, a no. 15 scalpel blade was utilized to create an oblique cutaneous groin incision under 16x magnification. For the motor nerve graft group, the motor branch to the quadriceps muscle was identified at its branch point from the femoral nerve. For the sensory nerve graft group, the femoral cutaneous branch was identified at its branch point from the femoral nerve. Under 25x magnification, for Groups 1 and 2, their respective nerves were neurolysed, resected, and divided into 5-mm segments for later use for reconstruction with sharp microscissors. The donor sites were re-approximated via simple interrupted 4-0 absorbable monocryl sutures in a subcuticular fashion. Tissue adhesive (3 M, Vetbond brand) was applied over the incision to assist with superficial reapproximation.

Then, under 16x magnification, a 2–3 mm post-auricular incision was fashioned via a no. 15 scalpel blade in an accommodating crease. After sharply dissecting through the underlying subcutaneous fascia, the main trunk of the facial nerve was identified at the level of the skull base. A micro Weitlaner retractor was utilized to obtain appropriate exposure. Upon appropriate exposure of the main trunk, under 25x magnification, the nerve was transected via sharp microscissors and a 5-mm gap was created. This step was performed for groups I, II, and III. For the control group, group III, this concluded the surgical intervention to be performed. For groups I and II, their respective autografts were introduced into the nerve gap and reapproximated in a tension-free fashion to the proximal and distal ends of the previously transected nerve under 40x magnification. Next, 11-0 nylon microsutures grasped in micro needle-drivers were used to attach each cable end to the nerve stump in an atraumatic fashion. Following this, the facial incision was closed in the previously described fashion with 4-0 subcuticular monocryl sutures and tissue adhesive. While anesthetized, the animals were given 0.5 mg/kg Carprofen injections for post-operative analgesia. Post-operative recovery was allowed to take place in an aseptic environment warmed via a heat lamp. The animals were closely observed for appropriate emergence from anesthesia. After their emergence, they were closely monitored for return of spontaneous activity of their four limbs as well as return of oral intake of soft diet gel. Following this, they were returned to the central animal care facility.

At the 15-week endpoint, the rats were again anesthetized in the aforementioned fashion. The animals’ left face was shaved and prepped as previously described. The left facial nerve was approached as described. The main trunk of the facial nerve, as well as the left marginal mandibular nerve (donor neve was harvested 1–2 mm proximal and distal to repair site), was resected with microscissors for histologic analysis. For group III, only distal segment main trunk was able to be harvested given that the injury was not repaired. The neural tissue was immersion-fixed in 4% paraformaldehyde. The facial incision was then extended toward the animals’ vibrissal follicles. The vibrissal musculature was then divided from the underlying tissue sharply via microscissors. The muscle specimen was cleaned, rinsed, flash-frozen in liquid nitrogen and then placed into a negative 80-degree freezer for further analysis. Following conclusion of the nerve and muscle harvest, the animals were euthanized in accordance with IACUC protocol.

### Electrophysiological analysis (Compound muscle action potential measurement)

At the 3, 5, 7, 9, 11, 13, and 15-week marks from surgery, electrophysiological data was obtained. Viking Quest Portable EMG (CareFusion, San Diego, California) software and hardware was utilized. Latency of signal responses as well as compound muscle action potential (CMAP) values were obtained from repetitive electrophysiological testing. CMAP amplitude values were calculated by measuring the magnitude between the maximum positive and negative turnaround point of the CMAP signal in millivolts (mV)^50^.

The rats were anesthetized as described above. Pain response was tested. Then, 28-gauge needle electrodes were placed proximally at the level of exit of the right facial nerve from the animal’s skull base. The sensing and reference electrodes were placed at a distance 4-mm from each other. The distal electrodes were placed to approximate the course of the marginal mandibular nerve, in close relation to the vibrissal pad. Again, the sensing and reference electrodes were placed at 4-mm distance from each other. A series of repetitive nerve stimulations was then performed, beginning at 10.0 mA and gradually increasing to the maximum allowed threshold of 24.8 mA. This repetitive stimulation generated a series of single square-wave pulses of 0.1 millisecond duration via the disposable monopolar electrodes. An identical procedure was performed for the opposite (left) side of the animal’s face.

### Muscle histomorphometric analysis

Harvested muscle was frozen in liquid nitrogen and stored in a −80 °C freezer until processing. Cross-sections (14-µm) were obtained from vibrissal muscles. The muscle sections were hydrated in distilled water for 5 minutes and placed in 20% sodium sulfate solution for 3 minutes. The sections were then rinsed in distilled water for 5 minutes and incubated in acetylcholinesterase solution (5‐bromoindoxyl acetate, 4.0 mg; ethanol, 0.3 mL; potassium ferrocyanide, 63.0 mg; potassium ferricyanide, 50.0 mg; Tris hydrochloric acid, 42.0 mg; Tris base, 4.0 mg; calcium chloride, 33.0 mg; and distilled water, 30.0 mL) for 75 minutes at 37 °C.

The sections were then rinsed with phosphate buffer solution (PBS) (11.9 mM phosphates, 137 mM sodium chloride, and 2.7 mM potassium chloride) for 5 minutes. Following the rinse, the sections were placed in 0.5% hydrogen peroxide solution in PBS for 10 minutes. The sections then underwent two rinses in PBS for 10 minutes each. The sections were blocked with 0.1% Triton-X 100 (Sigma, St. Louis, MO), 2% nonfat dry milk, and 1% normal goat serum in PBS.

Following the blocking solution, the sections received the primary antibody (polyclonal, neurofilament, 1:200, Cat# AB1987; Millipore, Houston, TX]) and were incubated at room temperature overnight. The following day, the sections underwent two rinses in PBS for 5 minutes each. The secondary antibody (anti‐rabbit IgG biotinylated, Vectastain ABC kit, Cat# PK4001; Vector Laboratories, Inc., Burlingame, CA) was applied for 60 minutes at room temperature. The sections were rinsed twice in PBS for 5 minutes each and processed with Vectastain ABC and DAB kits (Peroxidase substrate kit; Vector Laboratories, Inc.). The sections underwent two rinses in PBS and were dehydrated using ethanol and xylene (50% for 2 minutes, 70% for 2 minutes, 95% for 2 minutes [twice], 100% for 2 minutes [twice], and xylene for 10 minutes [twice]). The sections were then mounted with ProLong Gold antifade reagent (Invitrogen, Eugene, OR) and stored at −80 °C. Images were taken at 20x magnification.

### Nerve histomorphometric analysis

Harvested nerve was fixed in 4% paraformaldehyde and stored in a 4 °C refrigerator overnight. The nerves were then transferred to a 30% (w/v) sucrose solution in PBS and stored in a 4 °C refrigerator overnight. The nerves were then embedded in a cryomold with O.C.T. Compound (Fisher Healthcare, Waltham, MA) and were immediately frozen with dry ice and stored in a −80 °C freezer. Cross-sections of 14-µm were obtained from the embedded nerve samples using a Leica CM1850 microtome. Nerve sections were then stained with a neurofilament antibody.

The sections were blocked with 0.3% Triton-X 100 (Sigma, St. Louis, MO) and 5% bovine serum albumin in PB. Following the blocking solution, the sections received the primary antibody (polyclonal, neurofilament, 1:200, Cat# AB1987; Millipore, Houston, TX]) and were incubated at room temperature overnight. The following day, the sections underwent two rinses in PBS for 5 minutes each. The secondary antibody (donkey anti-rabbit Alexa Fluor 594, 1:1000, Cat# R37119; ThermoFisher Scientific, Waltham, MA) was applied for 60 minutes at room temperature. The sections were rinsed twice in PB for 5 minutes each. The sections were then mounted with ProLong Gold antifade reagent (Invitrogen, Eugene, OR).

Analysis took place via light microscopy at 20x magnification with an Olympus BX43 light microscope (Tokyo, Japan). Manual counts of nerve fibers were performed by an experienced lab member who was blinded to the experiment. This manual count was performed on four separate representative slides for each animal.

Fluorescent microscopy was performed at 20x magnification via Nikon Microphot FXA fluorescent microscope (Tokyo, Japan). Fluorescent microscopic images were then converted to digital images for quantification purposes. At 20x magnification, manual counts were performed by an experienced lab member to quantify the number of axonal fibers per 72 µm² square boxes33. This lab member was blinded to the experimental groups. The overall nerve architecture, quality, and quantity of the regenerated nerve fibers was assessed for each slide. This manual count was multiplied by the number of square boxes represented within the cross-sectional image to obtain an estimation of the total fibers present33. This was divided by the square area in µm to obtain nerve density measurement (axonal fibers/µm²). This value was determined for all the animals in our experimental groups and a mean was calculated for each cohort for direct comparative analysis.

### Nerve functional analysis

At identical time-points to performance of electrophysiological testing (3, 5, 7, 9, 11, and 13-weeks), functional analysis of whisker movements was performed^51,52^. Each individual animal was recorded in a transparent holding container for 30–60 seconds as needed to obtain prolonged assessment of vibrissal movement. As previously described, whisker movements were staged via a 4-point scale (0, no whisker movement, 1, slight whisker movement, 2, slow movement, 3, rapid movement undistinguishable from contralateral side)^52^. Eye movements were also staged via a 4-point scale (0, no perceptible motion, 1, muscle contraction but no perceptible motion, 2, incomplete eye closure, and 3, complete eye closure). Eye symmetry (0, asymmetric, 1, normal) and vibrissae symmetry at rest (0, asymmetric, 1, normal) were also assessed. At the end of the experiment, three blinded observers were asked to grade the whisker movements at random time-points to reduce likelihood of bias.

### Statistical analysis

Statistical analysis was performed via SPSS version 22 software (IBM; Armonk, NY). All results for quantitative analysis was reported as mean with standard deviation (error bars) represented. Power analysis was performed to determine the minimum sample size for α = 0.05, with a two-tailed t-test at a statistical power of 80 percent. Therefore, n = 6 per group was chosen to permit detection of differences in two means which, based on the literature, would be reasonable to expect of results from this experiment. Histomorphometric data was analyzed via student’s t-test and analysis of variations (ANOVA). All statistical tests of significance were two-sided with α of 0.05.

## Data Availability

All data generated or analyzed in this study are included in the published article.
